# The Development of Stimulus and Response Interference Control in Midchildhood

**DOI:** 10.1037/dev0000074

**Published:** 2015-11-23

**Authors:** Lucy Cragg

**Affiliations:** 1 University of Nottingham

**Keywords:** interference control, stimulus conflict, response conflict, flanker task, conflict adaptation

## Abstract

Interference control, the ability to overcome distraction from irrelevant information, undergoes considerable improvement during childhood, yet the mechanisms driving these changes remain unclear. The present study investigated the relative influence of interference at the level of the stimulus or the response. Seven-, 10-, and 20-year-olds completed a flanker paradigm in which stimulus and response interference was experimentally manipulated. The influence of stimulus interference decreased from 7 to 10 years, whereas there was no difference in response interference across age groups. The findings demonstrate that a range of processes contribute to the development of interference control and may influence performance to a greater or lesser extent depending on the task requirements and the age of the child.

Interference control, the ability to overcome distraction from irrelevant information, is a critical skill that is vital for success in carrying out plans and acquiring new knowledge and that is also vital in social situations. Interference control is one of a set of skills known as executive functions, which help us guide and control our thoughts and actions. Executive functions mature slowly throughout childhood and adolescence and have been linked to a variety of positive developmental outcomes, including successful academic achievement (Bull & Lee, 2014; Christopher et al., 2012; Cragg & Gilmore, 2014) as well as health and wealth in later life (Moffitt et al., 2011). Interference control has been found to play a key role in children’s school performance as well as their ability to attribute mental states to themselves and others and understand that these can differ (theory of mind). A number of studies have indicated that children who are better able to overcome distraction from irrelevant information also perform better on assessments of reading (e.g., Kieffer, Kukovic, & Berry, 2013), mathematics (e.g., St Clair-Thompson & Gathercole, 2006; Visu-Petra, Cheie, Benga, & Miclea, 2011), and theory of mind (e.g., Carlson, Moses, & Breton, 2002). The precise mechanisms by which interference control is linked to these abilities remain unclear, however. A greater knowledge of the processes involved in overcoming distractions, and how these change with age, will help further our understanding of these relations. This may in turn lead to the potential to capitalize on this knowledge in order to develop interventions to improve school outcomes and social understanding.

Interference control relies on a number of different cognitive processes acting in parallel (e.g., Egner, 2008; Notebaert & Verguts, 2008). For example, ignoring the interruption of e-mail notification alerts can be achieved by focusing attention on the task at hand so as to filter out the alert at a perceptual level (reducing stimulus interference) and/or by suppressing the habitual motor response to click on the e-mail icon (reducing response interference). Situations where distractions have to be ignored are modeled by tasks such as the classic Stroop (MacLeod, 1991) and Eriksen flanker paradigms (Eriksen & Eriksen, 1974). In these tasks, two different stimuli, or two aspects of a stimulus, prime different motor responses. This elicits both stimulus interference—perceptual or representational competition between relevant and irrelevant stimulus dimensions—and response interference—competition between motor responses. Typically, resolving this interference between relevant and irrelevant stimuli and responses results in a cost; reaction times (RTs) increase and response accuracy decreases (Eriksen & Eriksen, 1974; MacLeod, 1991). The effects of stimulus and response interference are thought to additively contribute to this cost. Studies separating the two have shown that decrements in adults’ performance are partly due to stimulus interference but mostly originate from response interference (e.g., Verbruggen, Notebaert, Liefooghe, & Vandierendonck, 2006; Wendelken, Ditterich, Bunge, & Carter, 2009; Wendt, Heldmann, Münte, & Kluwe, 2007).

Children’s responses are slowed to a greater extent by combined stimulus and response interference than adults (Li, Hämmerer, Müller, Hommel, & Lindenberger, 2009; van Meel, Heslenfeld, Rommelse, Oosterlaan, & Sergeant, 2012; Waszak, Li, & Hommel, 2010), yet it is not clear to what extent this is driven by interference between stimulus representations versus competing motor responses. One possibility is that children show a similar pattern to adults, experiencing greater response interference than stimulus interference. Developmental change in interference control may therefore be driven by improvements in detecting and suppressing response interference. Alternatively, children may have greater difficulty suppressing irrelevant perceptual information compared to adults, and developmental change may be driven by improvements in detecting and suppressing stimulus interference. These two alternatives are not mutually exclusive, and a third possibility is that improvements in control over both stimulus and response interference improve with age.

## Response Interference

Developmental improvements in control over competing responses would be consistent with a large body of literature demonstrating improvements in the ability to suppress competing or prepotent motor responses in midchildhood. Many interference tasks used with young children require only response interference; a single stimulus is presented at a time, and so there is no interference from competing stimuli. In some cases, response interference arises from the repetition of a simple response that becomes prepotent, or automatic. This then has to be completely suppressed (e.g., go/no-go and stop-signal tasks) or replaced with an opposite response (antisaccade tasks) on the presentation of a different stimulus. Some researchers have argued that this form of inhibition is implemented by a “brake” applied to motor processes, located in the right inferior frontal cortex (Aron, Robbins, & Poldrack, 2004, 2014). Others propose that, instead, the role of the prefrontal cortex is to maintain abstract task-relevant information, such as *when* to inhibit a motor response, and that the actual suppression of motor outputs arises from downstream projections to the subthalamic nucleus (Munakata et al., 2011). According to this account, problems suppressing a motor response could in fact arise from difficulties in maintaining task goals in working memory.

Performance on go/no-go, stop-signal, and antisaccade tasks undergoes considerable improvement during childhood (e.g., Bedard et al., 2002; Brocki & Bohlin, 2004; Cragg & Nation, 2008; Luna, Garver, Urban, Lazar, & Sweeney, 2004; Williams, Ponesse, Schachar, Logan, & Tannock, 1999), demonstrating increasing control over response-based interference with development. This suggests that developments in interference control are likely to be driven, at least in part, by improvements in control over response interference. This is also consistent with recent findings from stimulus–response compatibility paradigms such as the day–night Stroop task and the hand game (Simpson & Riggs, 2011), which also contain no stimulus–stimulus conflict. In these paradigms, children are required to give an opposite response to the stimuli presented—for example, to say *day* to a picture of the moon and *night* to a picture of the sun. Using careful experimental manipulations, Simpson et al. (2012) demonstrated that 3- to 4-year-olds have difficulty with this task due to the fact that the name of the stimulus is part of the response set rather than because it is semantically related to the correct response.

## Stimulus Interference

Developmental theories also propose improvements in the ability to maintain strong active representations of attention-guiding rules with age (e.g., Morton & Munakata, 2002; Munakata, Snyder, & Chatham, 2012). The representation of this abstract task-relevant information is thought to be a key function of the prefrontal cortex (Munakata et al., 2011), which then biases neural activity in goal-related processing areas. This consequently biases attention toward task-relevant stimulus features, allowing them to better compete with task-irrelevant information. This theory therefore implies that as the ability to strongly maintain attention-guiding rules improves during childhood, so does attentional control over stimulus interference. Evidence for this comes from a number of situations where participants have to ignore distractors that are not linked to specific motor responses. The Garner interference effect, where variation on an irrelevant dimension of a stimulus interferes with making speeded classifications on another dimension, is a classic measure of stimulus interference. When faced with the Garner paradigm, 6- and 7-year-olds display greater interference from irrelevant stimulus dimensions compared to adults (Shepp & Barrett, 1991; Strutt, Anderson, & Well, 1975). Performance on tasks that require searching for a target among distractors with similar features (i.e., conjunction search) also improves during midchildhood (e.g., Hommel, Li, & Li, 2004; Zhan et al., 2010). Moreover, recent fMRI studies recording brain activity while 7- to 13-year-olds and adults were asked to bias their attention to either face or scene images have demonstrated that children are less able to modulate cortical activity in brain areas related to stimulus processing (Vuontela et al., 2013; Wendelken, Baym, Gazzaley, & Bunge, 2011). Together, these results suggest that developmental improvements in control over stimulus interference also take place during midchildhood and may contribute to developmental changes in interference control when both stimulus and response interference are present.

## Stimulus Versus Response Interference

A number of previous studies have directly addressed developments in stimulus versus response interference in situations where both types of interference are present. Using a dual-mapping flanker paradigm, Enns and Akhtar (1989) found that 5- to 8-year-olds showed more interference from irrelevant stimulus information than from competing responses; however, this was related to the number of flankers present rather than to stimulus features. Other studies have used event-related potentials (ERPs) to index the amount of stimulus and response interference. The lateralized readiness potential (LRP) is an ERP component that reflects preparation of the correct versus the incorrect response in bimanual choice tasks. Developmental changes in the amplitude of the LRP (Szűcs, Soltész, Bryce, & Whitebread, 2009) and onset latency of correct response preparation (Ridderinkhof & van der Molen, 1995) have been interpreted as reductions in response interference with age. In a recent study, Bryce, Szűcs, Soltész, and Whitebread (2011) compared a number of measures taken from the LRP while 5-year-olds, 8-year-olds, and adults completed an animal Stroop task. Initial incorrect response preparation latency and duration did not differ between age groups, which Bryce et al. interpreted as reflecting mature stimulus interference control. In contrast, the transition from incorrect to correct response preparation when interference was present took longer in the 5- and 8-year-olds than in the adults. This was interpreted as developmental improvement in response interference control. Developmental changes in the P3, interpreted as reflecting sensitivity to stimulus interference, have shown developmental changes in some studies (Rueda, Posner, Rothbart, & Davis-Stober, 2004) but not others (Ridderinkhof & van der Molen, 1995; Szűcs et al., 2009). These studies indicate greater development of response than stimulus interference control. However, stimulus and response interference were not experimentally manipulated within these paradigms and, as such, it is difficult to separate out the influences of the two different sources of interference.

Studies in which stimulus and response interference are experimentally manipulated provide stronger evidence that stimulus interference may contribute to young children’s difficulty in situations that require interference control. Jongen and Jonkman (2008) asked 6- to 12-year-olds and adults to perform a fruit Stroop paradigm in which they had to name the printed color of a fruit. The printed color was either the same (congruent) or different from (incongruent) its canonical color, thereby creating stimulus interference. They found no developmental changes in stimulus interference. The size of the stimulus interference effect was very small at all ages, however, suggesting that the canonical color may not have been strongly primed by the fruit. Despite a lack of behavioral effects, 6- to 7-year-olds showed an amplitude enhancement of a negative (N4) component compared to older age groups. This component was interpreted as reflecting the detection of interference and the implementation of control and conflict resolution and suggests that 6- to 7-year-olds needed to recruit greater control over interfering stimulus information in order to match the behavioral performance of the older age groups. In a more recent study, Bossert, Kaurin, Preckel, and Frings (2014) used a version of the Eriksen flanker task in which they independently studied response interference in 7- to 12-year-olds while controlling for stimulus interference. The size of the flanker effect was smaller than in previous studies with this age group on tasks where both stimulus and response interference were involved. Moreover, Bossert et al. found no developmental change in overcoming response interference between 7 and 12 years. This suggests that in a standard flanker task, children experience both stimulus and response interference and questions whether the developmental improvements seen on the task are in fact driven by improvements overcoming response interference.

Further evidence that children experience interference from irrelevant stimulus properties comes from task-switching studies in which children are asked to switch between sorting images either by their color or shape while ignoring the other dimension. Manipulations of stimulus interference have been found to have a greater effect on children’s performance than manipulations of response interference (Cragg & Nation, 2009; Diamond, Carlson, & Beck, 2005; Rennie, Bull, & Diamond, 2004; Towse, Redbond, Houston-Price, & Cook, 2000). These findings suggest that children younger than 8 years may experience interference between stimuli in addition to response interference and highlight the need for further study into the role of stimulus interference in the development of interference control.

## Conflict Adaptation Effects

The standard measure on a flanker task to index interference control is the difference between performance on congruent trials, where no interference is present, and performance on incongruent trials, where interference from irrelevant stimuli and/or competing responses is present. This measure can be influenced by a number of factors, including the extent to which irrelevant information is processed, the time taken to detect the interference, and also the time taken to apply top-down control in order to resolve this interference and make a response. An additional measure, thought to specifically reflect the engagement of top-down control processes, is the Gratton, or conflict adaptation, effect. This refers to a reduced interference effect and faster performance on incongruent trials that are preceded by incongruent trials compared to incongruent trials preceded by congruent trials (e.g., Gratton, Coles, & Donchin, 1992). If top-down control processes are engaged following the detection of interference—for example, to increase the focus of attention to the target location, stimulus dimension, or correct response—then it is thought that this should spill over to improve performance on the subsequent trial, thereby reducing the interference effect.

Alternatively, conflict adaptation effects may be driven by associative priming of stimulus and response features (Hommel, Proctor, & Vu, 2004; Mayr, Awh, & Laurey, 2003). This view maintains that conflict adaptation effects are not driven by cognitive control but by episodic memory effects of stimulus–response associations. If a stimulus and response co-occur in time, they are linked together in a memory representation, such that a subsequent activation of one feature automatically activates the other. A complete repetition or alternation of the features results in faster performance than if only one of the features is repeated, but another is required to change, as the previous stimulus–response binding has to be overcome. This could explain conflict adaptation effects in a typical four-stimulus flanker task, as incongruent–incongruent trial sequences consist of complete repetitions or alternations of stimulus–response features and are therefore faster than incongruent–congruent sequences, which consist of partial alternations. Counter to this, the conflict adaptation effect remains when there are no repeats of stimulus and response features across trials (Liu, Chen, Li, Li, & West, 2012; Verbruggen et al., 2006), demonstrating that although associative priming may contribute to conflict adaptation effects, additional engagement of top-down control does occur.

In adults, the presence of response interference (e.g., Liu, Slotnick, Serences, & Yantis, 2003; Scerif, Worden, Davidson, Seiger, & Casey, 2006) has been shown to trigger top-down control, as seen in conflict adaptation effects. According to the conflict monitoring or conflict control loop theory (Botvinick, Braver, Barch, Carter, & Cohen, 2001), an influential model of response interference, the detection of response interference biases attention toward task-relevant stimuli or stimulus dimensions, resulting in less susceptibility to the irrelevant stimulus features, and therefore the irrelevant response, on the subsequent trial. Conflict adaptation effects following competing responses are evident in children as young as 4 years of age (Kray, Karbach, & Blaye, 2012), suggesting that children are able to apply top-down attentional control to adapt their behavior in the face of response interference. Yet the age at which this ability reaches adult levels is unclear. Some evidence suggests that it is mature by 8–11 years (Larson, Clawson, Clayson, & South, 2012), while other findings suggest that children are not successfully able to adapt top-down control in response to previous interference until 14 years of age (Waxer & Morton, 2011). Top-down control is also initiated following stimulus interference in adults (e.g., Egner, 2007; Verbruggen et al., 2006). To our knowledge, there are currently no studies investigating conflict adaptation effects following stimulus interference in children, however.

## The Current Study

**Figure 1 fig1:**
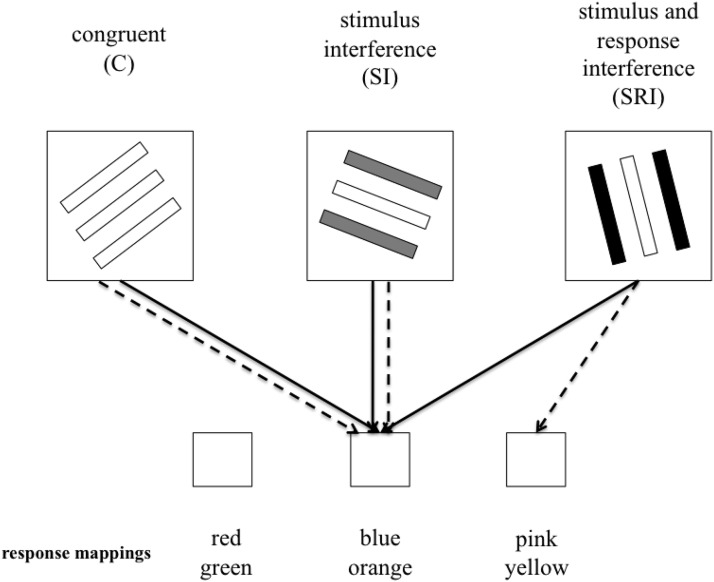
Examples of the stimulus conditions and the corresponding response mappings for the central target line (solid arrow) and flanker lines (dashed arrow). For the colors of the stimulus lines, white represents blue, gray represents orange, and black represents pink.

The current study used an experimental approach to compare developmental changes in the ability to overcome interference from irrelevant stimuli and competing motor responses. Seven-year-olds, 10-year-olds, and adults were asked to complete a modified version of the flanker task in which stimulus and response interference were experimentally manipulated. Participants were shown stimuli consisting of three parallel colored lines (see Figure 1) and were asked to indicate the color of the central line while ignoring the flanking lines. These flanking lines could either be the same color as the central line (congruent trials), a different color but mapped to the same response button (stimulus interference trials), or a different color mapped to a different response button (stimulus and response interference trials).[Fig-anchor fig1]

Two groups of children were included in order to be able to study changes within midchildhood rather than to simply compare children and adults. The two ages were chosen based on previous studies, which have demonstrated improvements in interference and inhibitory control between these ages (Brocki & Bohlin, 2004; Cragg & Nation, 2009; Jongen & Jonkman, 2008; Williams et al., 1999). This choice of age groups also facilitates comparison with previous studies that have investigated developmental changes in stimulus and response interference (e.g., Jongen & Jonkman, 2008). An adult group was also included as a reference point for mature performance. Due to developments in both control over competing motor responses (e.g., Bedard et al., 2002; Brocki & Bohlin, 2004; Cragg & Nation, 2008; Luna et al., 2004; Williams et al., 1999) and representation of attention-guiding rules (Hommel et al., 2004; Shepp & Barrett, 1991; Strutt et al., 1975; Vuontela et al., 2013; Wendelken et al., 2011; Zhan et al., 2010), we predicted that improved performance with age would be related to increases in overcoming stimulus interference as well as response interference.

Conflict adaptation effects were measured in order to investigate the extent to which children are able to exert top-down control over both stimulus and response interference. Based on previous findings, we predicted that both children and adults would show conflict adaptation effects to some extent following response interference (Kray et al., 2012; Larson et al., 2012; Liu et al., 2003; Scerif et al., 2006; Waxer & Morton, 2011). Adults were expected to show conflict adaptation effects following stimulus interference (e.g., Egner, 2007; Verbruggen et al., 2006), but no predictions were made regarding conflict adaptation effects following stimulus interference in children.

## Method

### Participants

Thirty-nine 7-year-olds (17 male, *M* = 6.91, *SD* = .49), thirty-five 10-year-olds (20 male, *M* = 10.3, *SD* = .33), and 38 young adults (11 male, *M* = 20.9, *SD* = 1.39) with normal or corrected-to-normal vision took part in the study. All participants were native English speakers with the exception of one native Chinese-speaking adult. The children were recruited from schools in Nottinghamshire, United Kingdom (UK), and were of average socioeconomic status (average Index of Deprivation according to school postcode = .53, where 0 = most deprived and 1 = least deprived). Written informed consent was given by each child’s parent or guardian, and the children themselves gave verbal assent. The children received a sticker at the end of the testing session as a reward for taking part. The young adults were students at the University of Nottingham and received either course credit or a small monetary inconvenience allowance for taking part. All adult participants gave written informed consent.

### Materials

The task was based on a paradigm used by Verbruggen et al. (2006). It was programmed in E-Prime (www.pstnet.com/eprime) and presented on a Samsung P510 laptop. The stimuli consisted of three parallel lines presented in the center of the screen inside a 1.9-cm square. The square remained on the screen throughout the experiment and served as the fixation point. The lines could be one of six colors (red, green, blue, orange, pink, or yellow). Each target line could be paired with a flanking line of (a) the same color (congruent condition [C]), (b) another color mapped to the same response (stimulus incongruent condition [SI]), or (c) another color mapped to a different response (stimulus and response incongruent condition [SRI]). The target was only paired with one response incongruent color so that all stimuli were presented an equal amount of times. The colors were selected on each trial so that there were no repetitions of color for targets and flankers and so that the flanker color could not become the target color, and vice versa, in order to eliminate associative priming across trials. The orientation of the three lines also varied randomly (selected from 10 possible orientations) so that a focusing strategy could not be used.

The participants’ task was to ignore the flanking lines and respond to the color of the central line by pressing a corresponding button on a RB-C30 Cedrus response pad (www.cedrus.com). Three buttons were used corresponding to the index, middle, and ring fingers of the right hand. The six colors were mapped onto the three buttons as follows: left button = red and green, middle button = blue and orange, right button = pink and yellow. Stickers with squares of color were placed on the keys as reminders of these response mappings.

### Procedure

The participants were tested individually in a quiet room at their school or university. The task began with 12 practice trials during which reminders of the response mappings were presented on the screen. This was followed by 12 trials without the reminders. During both of these blocks, feedback on accuracy was presented after each trial. The main task consisted of five blocks of 72 trials. The three congruence conditions (C, SI, and SRI trials) were presented equally often, resulting in 120 trials per condition. Taking into account congruency on the previous trial resulted in nine different transition types: C–C, C–SI, C–SRI, SI–C, SI–SI, SI–SRI, SRI–C, SRI–SI, and SRI–SRI. Adult participants were required to respond within 1,500 ms; however, pilot testing demonstrated that limiting RTs in this way was not appropriate for children. Instead, children were encouraged to respond as quickly as they could by presenting their completion time at the end of each block. A jittered interstimulus interval (ISI) of 750–1,250 ms was used, and a small red *oops* appeared underneath the stimulus for the first 200 ms of the ISI if an error was made.

## Results

The first five trials from each block and any trial on which the colors repeated across trials (0.04% of all trials) were excluded from data analysis. Incorrect trials and trials following an error were excluded from the RT analysis. Mean accuracy and median RTs were calculated for each participant for each transition type. Group means, standard deviations, and the number of included trials for all conditions are presented in Table 1. Degrees of freedom were corrected using Greenhouse–Geisser estimates of sphericity where necessary. Significant effects were followed up with tests of simple main effects and Bonferroni-corrected *t* tests comparing all conditions.[Table-anchor tbl1]

### Reaction Time

#### Median reaction time

There were differences in overall speed between the three age groups, with the fastest performance in the adults and the slowest performance in the 7-year-olds. There was also evidence of interference effects, with the fastest performance in the congruent condition and the slowest performance on the trials that contained both stimulus and response interference. A repeated-measures analysis of variance (ANOVA) with current congruence (C, SI, SRI) and previous congruence (C, SI, SRI) as within-subject factors and age group (7-year-olds, 10-year-olds, adults) as a between-subjects factor confirmed a significant main effect of age group, *F*(2, 109) = 114, *p* < .001, η_*p*_^2^ = .68. Post hoc comparisons revealed that this was due to overall slower performance for the 7-year-olds (*M* = 1,585, *SE* = 44) compared to the 10-year-olds (*M* = 983, *SE* = 46; *p* < .001, *d* = 2.19), who were slower than the adults (*M* = 651, *SE* = 45; *p* < .001, *d* = 1.21). The presence of interference effects was confirmed by a significant main effect of current congruence, *F*(1.49, 162) = 54.1, *p* < .001, η_*p*_^2^ = .332, which post hoc tests demonstrated was due to both significant response interference (slower performance on SRI trials [*M* = 1,159, *SE* = 35] compared to SI trials [*M* = 1,084, *SE* = 29; *p* < .001, *d* = 0.58]) and significant stimulus interference (slower performance on SI trials compared to C trials [*M* = 976, *SE* = 18; *p* < .001, *d* = 0.84]). The main effect of current congruence was qualified by a significant Age Group × Current Congruence interaction, *F*(2.98, 162) = 15.7, *p* < .001, η_*p*_^2^ = .224, suggesting that the size of the interference effects differed between the three age groups. There was also a marginally significant Current Congruence × Previous Congruence interaction, *F*(2.59, 282) = 2.69, *p* = .055, η_*p*_^2^ = .024, indicating conflict adaptation effects. Ratio scores were calculated for stimulus interference (SI:C) and response interference (SRI:SI) in order to explore these interactions further while controlling for differences in overall RT between the age groups. The same pattern of results was found when the analyses were rerun using difference scores. A ratio score greater than 1 demonstrates that interference slowed performance.

#### Ratio costs

**Figure 2 fig2:**
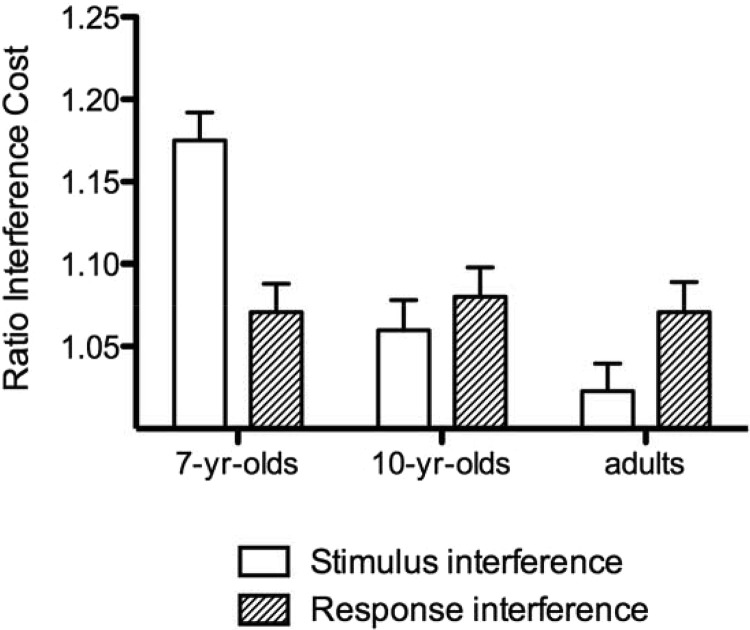
Stimulus and response interference ratio costs for reaction time (RT) in 7-year-olds, 10-year-olds, and adults.

**Figure 3 fig3:**
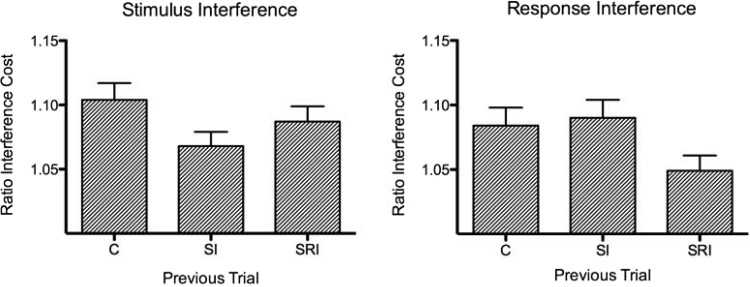
Stimulus and response interference ratio costs for reaction time (RT) as a function of previous trial congruency.

In order to determine whether stimulus and response interference effects had significantly slowed performance, a series of Bonferroni-corrected one-sample *t* tests were first performed to check that the ratio costs were significantly greater than 1. This confirmed significant effects of all types of interference at all age groups, with the exception of stimulus interference in adults (see Table 2). As shown in Figure 2, the pattern of interference effects differed for stimulus and response interference; whereas response interference effects were of a similar magnitude in all age groups, stimulus interference effects were much larger for the 7-year-olds than the 10-year-olds and adults. Moreover, 7-year-olds appeared to experience greater stimulus interference compared to response interference. A repeated-measures ANOVA was performed on the ratio costs with interference type (stimulus interference, response interference) and previous congruence (C, SI, SRI) as within-subject factors and age group (7-year-olds, 10-year-olds, adults) as a between-subjects factor. This revealed a significant main effect of age group, *F*(2, 109) = 10.1, *p* < .001, η_*p*_^2^ = .16. Post hoc comparisons demonstrated that this was due to overall greater interference for the 7-year-olds (*M* = 1.12, *SE* = .012) compared to both the 10-year-olds (*M* = 1.07, *SE* = .013; *p* = .01, *d* = 0.69) and adults (*M* = 1.05, *SE* = .012; *p* < .001, *d* = 1.05), who did not differ. There was no significant main effect of interference type, *F*(1, 109) < 1, *ns*; however, there was a significant Age Group × Interference Type interaction, *F*(2, 109) = 10.7, *p* < .001, η_*p*_^2^ = .16. Tests of simple main effects demonstrated that this was due to a significant effect of age group for stimulus interference, *F*(2, 109) = 21.5, *p* < .001, η_*p*_^2^ = .28, but not for response interference, *F*(2, 109) < 1, *ns*. Post hoc comparisons revealed significantly greater stimulus interference in 7-year-olds compared to 10-year-olds (*p* < .001, *d* = 1.07), but no difference between 10-year-olds and adults. A significant main effect of previous congruence, *F*(2, 218) = 6.66, *p* = .002, η_*p*_^2^ = .058, was qualified by a significant Previous Congruence × Interference Type interaction, *F*(2, 218) = 3.64, *p* = .030, η_*p*_^2^ = .032, indicating significant conflict adaptation effects. As shown in Figure 3, conflict adaptation effects appeared to be limited to the type of interference; that is, stimulus interference was reduced following a trial that contained only stimulus interference, and response interference was reduced following a trial that contained response interference. Post hoc comparisons confirmed a reduction in stimulus interference following SI trials compared to C trials (*p* = .006, *d* = 0.33) and a reduction in response interference following SRI trials compared to SI trials (*p* = .016, *d* = 0.28). The three-way interaction between age group, previous congruence, and interference type was not significant, *F*(4, 218) < 1, *ns*.[Table-anchor tbl2][Fig-anchor fig2][Fig-anchor fig3]

### Accuracy

A repeated-measures ANOVA with current congruence (C, SI, SRI) and previous congruence (C, SI, SRI) as within-subject factors and age group (7-year-olds, 10-year-olds, adults) as a between-subjects factor was performed. There was a significant main effect of current congruence, *F*(1.29, 140) = 59.2, *p* < .001, η_*p*_^2^ = .352, which post hoc tests demonstrated was due to less accurate performance on SRI trials (*M* = .90, *SE* = .008) compared to both C (*M* = .95, *SE* = .004; *p* < .001, *d* = 0.88) and SI trials (*M* = .96, *SE* = .005; *p* < .001, *d* = 0.89), which did not differ. This shows that accuracy was influenced by response interference but not stimulus interference. There was also a significant main effect of previous congruence, *F*(1.84, 201) = 4.15, *p* = .020, η_*p*_^2^ = .037, which was qualified by a significant Current Congruence × Previous Congruence interaction, *F*(2.99, 326) = 4.42, *p* = .005, η_*p*_^2^ = .039, indicating conflict adaptation effects. Tests of simple main effects showed that there was an effect of previous congruence on SRI trials, *F*(2, 108) = 6.08, *p* = .003, η_*p*_^2^ = .101, but not C trials, *F*(2, 108) < 1, *ns*, or SI trials *F*(2, 108) = 2.30, *ns*. Conflict adaptation effects on accuracy therefore also seemed to be restricted to response interference. Post hoc comparisons demonstrated that performance was more accurate on SRI trials when the previous trial was an SRI trial (*M* = .91, *SE* = .01) compared to an SI trial (*M* = .89, *SE* = .01; *p* = .004, *d* = 0.27) or a C trial (*M* = .90, *SE* = .01; *p* = .052, *d* = 0.24); that is, response interference was reduced following a trial that also contained response interference. There was a significant main effect of age group, *F*(2, 109) = 30.6, *p* < .001, η_*p*_^2^ = .36, due to less accurate performance for the adults (*M* = .88, *SE* = .008) compared to both the 7-year-olds (*M* = .96, *SE* = .008; *p* < .001, *d* = 1.44) and 10-year-olds (*M* = .96, *SE* = .009; *p* < .001, *d* = 1.58), who did not differ. There were no further interactions involving age group.

## Discussion

This study aimed to elucidate the mechanisms of developmental change in interference control by investigating the relative influence of stimulus and response interference as well as the extent to which they were influenced by top-down control processes, as evidenced in the conflict adaptation effects. Seven-year-olds, 10-year-olds, and adults were required to indicate the color of a central colored line while ignoring flanking lines that were either the same color as the central line (congruent trials), a different color but mapped to the same response button (stimulus interference trials), or a different color mapped to a different response button (stimulus and response interference trials). Stimulus interference contributed significantly more to interference effects in 7-year-olds compared to 10-year-olds and adults, whereas response interference did not change with age. Both types of interference were subject to conflict adaptation effects, indicating top-down control adjustments in all age groups.

### Interference Effects

All participants showed a significant overall interference effect, with a slower and less accurate performance on trials containing interference. This demonstrates that, at any age, an individual’s behavior is influenced by interference from irrelevant information. There was a reduction in the overall RT interference effect with age between 7 and 10 years, demonstrating a developmental improvement in interference control. There was no difference in speed between 10-year-olds and adults, however, suggesting that interference control has already matured by this age. Other studies have found that improvements in interference control continue into adolescence (e.g., Li et al., 2009; Waszak et al., 2010); however, many of these studies failed to control for baseline differences in RT, which can mean that developmental differences are overinflated. Perhaps surprisingly, the adults were less accurate overall than the 7- and 10-year-olds. This was taken as a reflection of the fact that the adults were given a more stringent RT limit than the children. However, as there was no interaction with the experimental manipulation, this indicates that the slight difference in task between the children and adults did not have an adverse effect on the results.

#### Stimulus interference

Both groups of children, but not the adults, experienced significant stimulus interference from irrelevant distractors. Moreover, there was a developmental change, with 7-year-olds experiencing greater stimulus interference than 10-year-olds. This finding is consistent with the findings of Enns and Akhtar (1989) and developmental task-switching studies where developmental improvements in perceptual interference in childhood have also been found. It is also consistent with theoretical models of development that attribute improvements in top-down control to an increasing ability to maintain strong active representations of attention-guiding rules (e.g., Morton & Munakata, 2002; Munakata et al., 2012). The current findings are somewhat discrepant from studies that have compared the development of stimulus and response interference using ERPs, however. The majority of these studies did not experimentally manipulate stimulus and response interference, but used the latency of the P3 ERP component (Ridderinkhof & van der Molen, 1995; Szűcs et al., 2009) or the duration of the initial deflection of the incongruent LRP (Bryce et al., 2011) as an index of stimulus interference. It has been shown that the N450 may be a more consistent marker of stimulus interference, however (Szűcs & Soltész, 2012; West, Bowry, & McConville, 2004), and therefore changes in stimulus interference may have been overlooked in some studies. Consistent with this, Jongen and Jonkman (2008) demonstrated an effect of stimulus interference in the ERP between 400 and 560 ms in 6- to 7-year-olds, suggesting that this age group may have had to exert greater control to match the behavioral performance of the other groups.

A further explanation as to why this study found changes in stimulus interference whereas others have not is because differences in the specific task requirements may influence the extent to which stimulus interference effects are seen. In the current task, the flanking stimuli were presented extremely close to the target and the orientation of the stimuli changed on each trial, such that a focusing strategy on the target location could not be used. Moreover, color information has been found to be particularly salient to children around the age of 7 years (Cragg & Nation, 2009; Ridderinkhof, van der Molen, Band, & Bashore, 1997). A further difference to the standard flanker paradigm is that in the current task, two thirds of the trials (C and SI) were response compatible, with both the target and flankers indicating the same response, compared to half the response-compatible trials in standard flanker tasks. This manipulation may have led to participants paying more attention to the flankers in this study, as they are facilitative, rather than interfering on the majority of trials. It is also plausible that the flankers had a facilitative effect on the stimulus interference condition in the 10-year-olds and adults, who had learned the task set and color mappings well. In contrast, for the 7-year-olds, who may have had difficulty maintaining the task set in working memory, the flankers may not have had a facilitative effect, leading them to respond more slowly than the other groups in the stimulus interference condition. Taken together, these task differences may have resulted in larger stimulus interference effects than have been seen in other developmental studies comparing stimulus and response interference using other tasks.

#### Response interference

In contrast to previous studies comparing developmental changes in stimulus and response interference, there were no changes in the amount of response interference experienced between the age of 7 years and adulthood. It is possible that this is due to task differences between studies. In most developmental studies, there are typically a maximum of four stimuli mapped onto two different responses, and participants are required to memorize the stimulus–response mappings (e.g., Jongen & Jonkman, 2008; Konrad et al., 2005; van Meel et al., 2012; Waszak et al., 2010). In this study, six stimuli were mapped onto three different responses. Because of this added complexity, reminders of the stimulus–response mappings were constantly present, so it was not necessary for participants to memorize them. As such, competing responses may not have been automatically triggered by the flanking stimuli, particularly in the 7-year-olds, who may have had the most difficulty learning the stimulus–response mappings. However, in a recent study by Bossert et al. (2014), 7- to 12-year-olds completed a flanker task in which all trials contained stimulus interference so that the effects of response interference could be studied independently. Their task used simpler stimulus–response mappings (two colors per response) and also included an extensive practice session so that the children could learn the mappings. Despite this, no developmental change in response interference was found between the ages of 7 and 12 years. This suggests that the lack of developmental change in response interference in the current study was not simply due to difficulty learning the stimulus–response mappings and supports the finding that response interference undergoes little change in this age range. This is also consistent with results from stimulus–response compatibility tasks that find that control over competing responses improves dramatically in early childhood between 3 and 5 years but at a much slower rate during midchildhood (Simpson & Riggs, 2005).

### Conflict Adaptation Effects

Conflict adaptation effects, the influence of interference on performance on the subsequent trial, were studied in order to separate the top-down control of interference from its detection and determine if the presence of stimulus conflict is sufficient to trigger the top-down control of further interference. It has been suggested that conflict adaptation effects could arise simply from associative priming of stimuli and responses (Hommel et al., 2004; Mayr et al., 2003). In order to control for this, the present study used six colors mapped onto three responses so that there were no repeats of stimulus and response features across trials. Conflict adaptation effects were still seen in the data, suggesting that they do—at least in part—reflect top-down control processes rather than associative priming. The conflict adaptation effects did not interact with age, suggesting that all age groups were able to reduce interference by the same amount. This is consistent with the findings of Larson et al. (2012), who showed no age differences in conflict adaptation, but not with the results of Waxer and Morton (2011), who found poorer top-down control in 9- to 11-year-olds compared to adolescents and adults. The discrepancy in findings may be due to the more complex task used by Waxer and Morton, which involved task switching in addition to interference control.

#### Stimulus interference

Stimulus interference was significantly reduced following trials that contained only stimulus interference; that is, SI but not SRI trials. This suggests that stimulus interference is under top-down control and that the detection of stimulus interference results in an increase of attention toward the central target location, which persists onto the subsequent trial. As stimulus interference was also present on SRI trials, it would be expected that stimulus interference would also be reduced following these trials. The stimulus interference effect was slightly reduced following SRI trials compared to congruent trials, but this did not reach significance. This result is difficult to explain, but it may be that when both stimulus and response interference are present, resources also have to be allocated to top-down suppression of the competing response, and so top-down attentional control is reduced.

Although stimulus interference was reduced by the same extent in all three age groups following SI trials (a 0.04 reduction in the SI ratio), this had differing effects on stimulus interference in the three groups due to the overall differences in stimulus interference. In adults, who had an average stimulus interference ratio of 1.02, any reduction in stimulus interference reduces this value below 1, thereby removing stimulus interference completely. However, as the 7-year-olds had a much higher average stimulus interference ratio of 1.17, the ratio was reduced by the conflict adaptation effects but not eliminated. One way to explain these findings is within the framework of proactive and reactive control (Braver, 2012), where proactive control reflects the sustained and anticipatory maintenance of goal-relevant information, and reactive control reflects transient stimulus-driven goal reactivation based on interference demands. Children may be able to recruit reactive control processes to the same extent as adults in the face of stimulus interference, as seen in the conflict adaptation effects; however, they may not exert the same level of proactive control to limit initial interference from irrelevant stimulus information. Again, this is consistent with theories that propose developmental improvements in an increasing ability to maintain strong active representations of attention-guiding rules (e.g., Morton & Munakata, 2002; Munakata et al., 2012). Previous studies have suggested that children as young as 6 years are able to implement proactive control under certain conditions (Chatham, Frank, & Munakata, 2009); however, they do not always choose to do so (Chevalier, Martis, Curran, & Munakata, 2015). This seems to be the case for the 7-year-olds in this study, who did not appear to proactively prepare for stimulus interference in advance.

#### Response interference

Response interference was reduced following a trial that contained response interference, but not following one that contained stimulus interference alone. This pattern of findings suggests that control over responses is relaxed after trials where two sources of information lead to the same response, whereas when there is conflict between two responses, it is increased. The SRI trials were not significantly different from congruent trials, however, and therefore these results need to be interpreted cautiously. The different patterns of conflict adaptation effects seen for stimulus and response interference, with the detection of each type of interference only triggering increased control over that same type of interference, are suggestive of independent cognitive and neural mechanisms for implementing top-down control over stimulus and response interference. This is consistent with findings from the adult literature, which suggests that stimulus and response interference are detected by different areas of the anterior cingulate cortex (Kim, Kroger, & Kim, 2011; Milham & Banich, 2005; Venkatraman, Rosati, Taren, & Huettel, 2009) and subsequently recruit different networks of brain areas for top-down control (e.g., Nigbur, Cohen, Ridderinkhof, & Stürmer, 2012; Wendelken et al., 2009).

### Conclusions and Implications

This study demonstrates that improvements in the top-down control of stimulus interference contribute to developmental changes in interference control during midchildhood. Taken at face value, these results suggest that improvements in interference control in this age range are due to developments in attentional control over interfering stimuli rather than between competing responses. Put another way, in 7-year-olds, this flanker task created a significant amount of stimulus interference, which was not experienced to such a great extent by 10-year-olds and adults. These findings are, of course, cross-sectional, and future longitudinal studies are required in order to confirm this pattern of developmental change. Critically, however, this study demonstrates that interference control tasks measure a range of different processes, which may influence performance to a greater or lesser extent depending on the task requirements and the age of the participants.

These findings have important implications for studies that use interference control tasks to study changes in the organization of executive functions with age or the contribution of executive function skills to other areas of development, such as reading or mathematics. Being aware of these nuances in performance across tasks and ages may help us better understand these relations in more detail. A similar approach could also aid our understanding of atypical development. Deficits on interference control tasks are apparent in a range of developmental disorders, yet it may be that difficulties arise at different levels of processing in different groups.

## Figures and Tables

**Table 1 tbl1:** Group Means and Standard Deviations for All Conditions

	Accuracy *M* (*SD*)			Reaction time Mean of medians (*SD*)			Mean number of trials contributing to reaction time analysis (*SD*)		
Condition	7-year-olds	10-year-olds	Adults	7-year-olds	10-year-olds	Adults	7-year-olds	10-year-olds	Adults
Congruent trial	.98 (.03)	.98 (.02)	.90 (.06)	1,374 (290)	919 (130)	619 (71)	104 (7)	104 (5)	88 (13)
Previous trial: C	.98 (.04)	.98 (.03)	.90 (.07)	1,369 (288)	909 (126)	613 (74)	33 (5)	32 (5)	29 (5)
Previous trial: SI	.98 (.04)	.98 (.02)	.89 (.07)	1,381 (289)	925 (141)	617 (72)	39 (5)	38 (3)	31 (6)
Previous trial: SRI	.98 (.04)	.98 (.03)	.90 (.06)	1,385 (300)	925 (139)	627 (69)	32 (5)	33 (4)	28 (6)
Stimulus incongruent only trial	.98 (.04)	.98 (.01)	.91 (.07)	1,629 (506)	973 (159)	628 (69)	104 (7)	105 (5)	90 (13)
Previous trial: C	.98 (.03)	.99 (.02)	.92 (.06)	1,658 (554)	988 (165)	636 (73)	39 (5)	38 (4)	32 (5)
Previous trial: SI	.97 (.05)	.98 (.02)	.90 (.08)	1,606 (523)	964 (162)	620 (67)	42 (5)	44 (4)	37 (7)
Previous trial: SRI	.98 (.03)	.98 (.03)	.91 (.08)	1,645 (499)	975 (167)	636 (77)	24 (5)	23 (5)	20 (4)
Stimulus and response incongruent trial	.92 (.09)	.93 (.07)	.85 (.09)	1,716 (507)	1,053 (256)	671 (65)	96 (14)	99 (11)	83 (14)
Previous trial: C	.92 (.10)	.94 (.08)	.84 (.11)	1,757 (559)	1,069 (274)	690 (71)	32 (6)	34 (5)	27 (7)
Previous trial: SI	.91 (.10)	.91 (.12)	.85 (.10)	1,779 (651)	1,052 (259)	670 (77)	23 (4)	22 (4)	21 (4)
Previous trial: SRI	.93 (.10)	.94 (.06)	.86 (.08)	1,684 (547)	1,045 (257)	662 (67)	42 (9)	43 (7)	36 (7)
*Note.* C = congruent trial; SI = stimulus incongruent only trial; SRI = stimulus and response incongruent trial.									

**Table 2 tbl2:** Statistics for One-Sample t Tests of Interference Costs

Age group	*M*	*SD*	*t*	*p* (significance level controlling for multiple comparisons = .008)	Effect size (Cohen’s *d*)
7-year-olds					
Stimulus interference (SI:C)	1.17	.17	6.45	<.001	1.03
Response interference (SRI:SI)	1.07	.10	3.72	.001	.60
10-year-olds					
Stimulus interference (SI:C)	1.06	.05	7.00	<.001	1.18
Response interference (SRI:SI)	1.08	.14	3.39	.002	.57
Adults					
Stimulus interference (SI:C)	1.02	.05	1.18	.246	.19
Response interference (SRI:SI)	1.07	.06	7.51	<.001	1.22
*Note.* C = congruent trial; SI = stimulus incongruent only trial; SRI = stimulus and response incongruent trial.
